# 4021 Learning from Patient Experience to Improve Diagnosis: a Pilot Study

**DOI:** 10.1017/cts.2020.409

**Published:** 2020-07-29

**Authors:** Kelly Gleason, Cheryl Dennison Himmelfarb, Susan Peterson, Taylor Wynn, Mariel Villanueva, Paula Bondal, David Newman-Toker

**Affiliations:** 1 Johns Hopkins University School of Medicine

## Abstract

OBJECTIVES/GOALS: Leveraging Patient’s Experience to improve Diagnosis (LEAPED) is our proposed method of measuring diagnostic error through seeking patient feedback on their understanding of their diagnosis and health status following emergency department discharge. To pilot test LEAPED’s feasibility, we deployed and determined patient uptake of LEAPED. METHODS/STUDY POPULATION: To test LEAPED, we employed a longitudinal cohort study design at emergency departments across one academic health system in the Mid-Atlantic region. Patients consented to complete questionnaires regarding their understanding of their diagnosis and/or follow-up steps and their health status at 2 weeks, 1 month, and 3 months following emergency department discharge. People aged 18 and older who were seen at the emergency department within the past 7 days with at least one chronic condition (hypertension, diabetes, history of stroke, arthritis, cancer, heart disease, osteoporosis, depression, and/or chronic obstructive lung disease) and one or more of the following common chief complaints: chest pain, upper back pain, abdominal pain, shortness of breath/cough, dizziness, and headache were eligible to join the study. RESULTS/ANTICIPATED RESULTS: Of those enrolled (n = 59), 95% (n = 53) responded to the two week post-ED discharge questionnaire (1 and 3-month ongoing). Of the 6 non-responders, 1 had died and 3 were hospitalized at two weeks. The average age was 50 years (SD 16) and 64% were female. Over half of participants (53%) were white and 41% were black. Almost one-third (27%) reported they were not given an explanation of their health problem on leaving the ED, and of those, a third did not have an understanding of what steps to take after leaving the ED. Participants reported a new health problem was identified after ED discharge (19%), worsening health status (12%), and health status stayed the same (16%). DISCUSSION/SIGNIFICANCE OF IMPACT: Patient uptake of LEAPED was high, which suggests that patient-report is a feasible method of evaluating diagnostic decision making and delivery to patients and yields insightful information beyond administrative data. The next steps are to validate the accuracy of patient-reported diagnostic error by comparing with administrative data.

